# Sex as a Biological Variable in Early-Phase Oncology Clinical Trials: Enhancing the Path to Personalised Medicine

**DOI:** 10.1016/j.heliyon.2024.e32597

**Published:** 2024-06-07

**Authors:** Lydia Sutherland, Louise Carter

**Affiliations:** a Division of Cancer Sciences, School of Medical Sciences, University of Manchester, Manchester, UK; b Department of Pharmacy, The Christie NHS Foundation Trust, Manchester, UK; c Department of Medical Oncology, The Christie NHS Foundation Trust, Manchester, UK

## Abstract

Sex is an essential biological variable that influences the development, progression and response to treatment in cancer. Despite this, early-phase cancer clinical trials frequently neglect to consider sex as a variable, creating a barrier to the development of personalised medicine. This article argues that failure to identify and infer sex differences in early-phase clinical trials may result in suboptimal dosing, underestimation of toxicity, and the failure to identify potential sex-specific responses to new systemic anticancer therapies. There should be a greater focus on sex as a biological variable in drug development so that thoughtful and deliberate study design can bring precision to the development of new systemic cancer therapies.

## 1 Introduction

Personalised medicine aims to improve drug efficacy and minimise side effects by tailoring treatment to specific patient populations. The last decade has seen profound changes in oncology drug development, from generalised, organ-centric approaches to the precision targeting of tumour-specific molecular aberrations [1]. This emphasis on genomics has led to significant improvements in patient outcomes; however, failure to consider broader patient characteristics risks overlooking the impact of other complex modulators of disease and treatment response.

This oversight is especially striking when it comes to sex – a fundamental biological variable and determinant of health and disease throughout a patient's life [2]. Sex is a significant modulator of the endpoints assessed in phase 1 and 2 clinical trials [3], which evaluate the safety, tolerability, pharmacokinetics, pharmacodynamics and preliminary efficacy of new cancer therapies. During these early-phase trials, a maximum tolerated dose (MTD) is determined by administering increasing drug doses to a small group of patients. This information guides dose selection for testing in larger, phase 3 registrational trials. However, this paradigm relies on the assumption that “one dose fits all”; most cytotoxic agents and antibodies are dosed according to body surface area (BSA) and body weight, respectively. Similarly, targeted agents and some checkpoint inhibitors are administered at flat doses. However, pharmacokinetic differences in the rates of absorption, distribution and elimination means that the MTD of some drugs is likely to be lower in women than in men, and the administration of standard doses can lead to increased drug exposure and toxicity [4]. Conversely, the lower rates of toxicity in men receiving cytotoxic agents could indicate relative underdosing, potentially contributing to their poorer treatment outcomes [5].

The overall impact of these differences is substantial. Data suggests that women have a 1.5- to 2-fold greater risk of experiencing adverse drug reactions across all drug classes and are significantly more likely to be hospitalised due to reactions [4,6]. Moreover, off-target effects can lead to dose interruptions and reduced compliance. In the United States (US), the annual cost of adverse drug reactions alone is estimated to be $30.1 billion [7]. Early-phase studies should aim to build a more comprehensive foundation for dosing decisions to enable appropriate dose modifications based on patient-specific characteristics, including sex.

The limited inclusion of female animals in preclinical studies [8,9], along with the low participation of women in clinical trials [10,11] and the pooling of study results [12,13], has hindered our understanding of how biological sex influences treatment outcomes in oncology. Various regulatory agencies in the US, Europe and Canada have made some positive but fragmented efforts to incorporate sex into pharmaceutical regulations [14]. Additionally, international collaborations like the European Gender Medicine project aim to develop a strategic plan for integrating sex-related aspects into medical research [15]. Recently, there has been an increasing interest in the influence of sex hormones on pharmacodynamic responses to targeted agents [16] and immunotherapies [17,18]. Researchers have also identified sex-biased gene expression signatures in clinically actionable genes across several tumour types [19], underscoring the importance of developing treatment strategies specific to each sex. Despite these advancements, incorporating sex as a biological variable into early-phase clinical trial methodology remains limited due to conceptual and practical challenges, such as motivation, study design, data analysis and interpretation. In this review, we contend that including sex as a biological variable in the early stages of clinical drug development is a vital component that can considerably refine and improve the precision of new cancer therapies.

## 2 Sex matters: the need for sex-specific therapeutic approaches

### 2.1 Sex-specific variations in cancer development and therapeutic targets

The two fundamental differences between male and female cells are their sex chromosomes and the levels of sex hormones they are exposed to. The interplay between sex chromosomes and sex hormones influences both local determinants of carcinogenesis, such as cancer initiating cells and the tumour microenvironment, and systemic determinants, such as cell metabolism and the immune system [20]. Phenotypic differences based on sexual genotype (XX in females, XY in males) can arise via several mechanisms, including the presence or absence of single or double copies of the gene, genetic imprinting, meiotic effects and X-chromosome inactivation [2]. Indeed, the X chromosome has at least 23 tumour suppressor genes, with at least 8 genes that escape X-chromosome inactivation, conferring some protection against carcinogenesis in female patients [21]. In males, extreme downregulation or loss of the Y chromosome (LOY) has a strong association with 12 major non-reproductive cancers [22]. In the context of pancreatic cancer, where LOY occurs in ∼40 % of male cases, the development of squamous-like tumours requires the loss of both X and Y homologues [23]. Conversely, bi-allelic loss of the X-linked KDM6A, albeit rare, predicts poor outcomes in females with squamous-like pancreatic cancer and confers sensitivity to BET inhibitors, highlighting the need for sex-specific biomarkers and tailored treatment strategies [24].

Current genome-wide association studies often exclude sex chromosomes because existing analytical tools are not adequately equipped to address their unique technical challenges [23]. This has led to the preferential study of autosomes and sex disparities in cancer outcomes remaining poorly understood at a genetic level. However, comprehensive molecular analyses across multiple tumour types have identified extensive sex-biased patterns in autosomal gene expression, mutation patterns and loads [25]. Importantly, 53 % of clinically actionable genes showed sex-biased signatures [26]. This suggests that the development of predictive biomarkers based on unannotated data from both sexes may be skewed toward the sex more commonly affected by the specific tumour type. Unlike reproductive cancers, data on the effects of sex hormones and their receptors—including oestrogen receptors alpha (ERα) and ERβ, and the androgen receptor —on non-reproductive cancers are limited. Nevertheless, some insights have been gained. In squamous cell carcinoma (SCC), for instance, loss-of-function mutations or down-modulation of *NOTCH1* are known to impede normal squamous cell differentiation. ERβ directly regulates *NOTCH1* transcription during cell differentiation by promoting the resumption of RNA polymerase II activity, a process often disrupted in SCC of the head and neck, skin, and lungs. Experimentally increased ERβ expression or stimulation with ERβ agonists in SCC cells has been shown to inhibit cell proliferation and promote differentiation by inducing *NOTCH1* transcription and function [27]. Furthermore, studies in melanoma have shown ERβ expression levels to be inversely related to primary tumour progression in both men and women [28]. In mouse melanoma, treatment with tamoxifen, which may act as an ERβ agonists, significantly inhibited metastatic spread in females, although this effect was not tested in males [29].

The interaction between the immune system and cancer under the influence of sex is a developing research area. Generally, females mount stronger acute immune responses to both foreign and self-antigens, reducing cancer risks associated with chronic infection-associated inflammation whilst increasing the risk of many autoimmune diseases [30]. Sex differences in the number and function of immune cells are evolutionarily conserved across species from fruit flies to humans, partially attributable to the localisation of immune regulatory genes and micro-RNAs to the X-chromosome [31,32]. Bi-allelic expression of Toll-like receptor 7 (TLR7), for example, is essential for key immune cell activation and pathogen recognition, and escapes X-chromosome silencing in female cells [33]. Similarly, activation of the X-linked TLR8 signalling in regulatory T cells (T_reg_) may impair immunosuppression by selectively inhibiting glucose uptake and subsequently triggering their senescence. This in turn reduces the suppression of effector T cells by T_reg_ and establishes a fundamental X-linked adaptive immune-metabolic axis [34]. TLR8 agonists that are currently being investigated as new cancer immunotherapies [35] may therefore be particularly beneficial in male patients due to their singular X-chromosome expression [36].

Sex hormones constitute another major determinant of sex differences in anticancer immune response. Progesterone has extensive anti-inflammatory effects, oestradiol enhances cell-mediated and humoral responses, whilst androgens suppress immune cell activity [37]. Putative androgen response elements and oestrogen response elements are present in the promoter regions of several innate and adaptive genes, suggesting that sex hormones directly regulate their expression [30]. Preclinical studies suggest that 17β-oestradiol (E2), the main oestrogenic hormone, induces immunomodulation via enhanced expression of the PD-1 costimulatory pathway, potentiating T_reg_ cells and indirectly reducing effector T cell activation [38]. Tumour expression levels of PD-L1 have also been shown to be modulated in an E2-dependent and sex-specific manner across a large spectrum of cancers [30,39]. These differences are postulated to contribute to the observed sex differences in cancer prevalence and mortality [40,41], and the ramifications for immune checkpoint inhibitors targeting PD-1 and PD-L1 is a growing area of study [42]. Overall, these insights underscore the importance of considering sex-specific factors in patient selection for clinical trials and in developing tailored treatment strategies.

### 2.2 Sex differences in pharmacodynamics and efficacy of anticancer drugs

Pharmacodynamic differences between the sexes occur when the same circulating plasma concentration of a drug yields a different pharmacological response. Signalling pathways are believed to be similar in structure between the sexes, but their activity and regulation are influenced differently by sex hormones [43]. The vascular endothelial growth factor (VEGF)-targeting antibody bevacizumab, for example, confers less benefit to females with non-small cell lung cancer (NSCLC) undergoing chemotherapy compared to males, demonstrating efficacy in a sex- and dose-dependent manner [44]. Murine models indicate that oestrogen promotes marked resistance to bevacizumab in NSCLC by enhancing vascular pericyte coverage and myeloid infiltration, which was successfully reversed by the addition of the ER-antagonist fulvestrant [45]. This suggests that blocking oestrogen could enhance the efficacy of VEGF inhibitors in female NSCLC patients, where treatment resistance is a significant challenge. Conversely, females demonstrate significantly better progression-free survival with BRAF/MEK-targeted therapies than males [46]. Sustained androgen receptor signalling is associated with a poorer prognosis in human melanoma samples and promotes resistance to BRAF/MEK-targeted therapies [47,48]. Androgen blockade using enzalutamide has recently been shown to improve tumour control in both sexes treated with BRAF/MEK inhibitors [49], suggesting a potential strategy for improving outcomes across several cancers that are now being treated with these agents. In addition, androgen signalling promotes CD8^+^ T-cell exhaustion in tumour microenvironments, affecting the efficacy of immune checkpoint inhibitors [50,51]. Androgen receptor blockade enhances the response to *anti*-PD-1 immune checkpoint therapies in preclinical models, and clinical trials combining androgen blockade with BRAF/MEK-targeted therapies or immune checkpoint inhibitors are already underway [52].

Empowering immunity against cancer is core to a new wave of immunotherapies, yet functional differences in immunity between the sexes have been largely overlooked in clinical practice. Studies indicate that men derive considerably greater survival benefits from monotherapy using *anti*-CTLA4 and *anti*-PD-1 antibodies across several tumour types compared to women [53,54], where immune response is already high [30,55]. In contrast, women experience higher survival benefits from the addition of chemotherapy to *anti*-PD-1/PD-L1 antibodies, likely due to its ability to increase the mutational burden and neoantigenic load of female tumours, which are statistically significantly lower than in male tumours [42,56]. In male patients, the focus should therefore be on enhancing the immune environment, while in females, increasing tumour antigenicity could be more beneficial.

Targeting metabolic adaptations of cancer cells is a promising therapeutic approach, but sex differences in cancer metabolism should be considered. In NSCLC, the addition of the antifolate agent pemetrexed as a first-line treatment in combination with platinum compounds has improved survival and overall response rates; however, about 50 % of patients with advanced disease still show no response to treatment [57,58]. Recent research into the differential metabolic pathways of NSCLC cells has shown increased de novo serine and glycine biosynthesis from glucose to predict increased pemetrexed-induced cytotoxicity in male, but not female, cell lines [59], suggesting that sex-specific analyses of predictive metabolic biomarkers may help stratify patients for treatment approaches. Similarly, elevated blood glucose during chemotherapy is associated with increased chemoresistance [60]. Studies of colorectal adenocarcinoma cells have found that prolonged exposure to high glucose promotes resistance to doxorubicin and 5-fluorouracil by reducing the production of treatment-induced mitochondrial reactive oxygen species to levels below what is necessary to induce apoptosis [61]. Given that males have higher average blood glucose levels [62], they may also be at disproportionate risk of developing chemoresistance.

Sex differences in the pharmacodynamic sensitivity to certain cancer therapies cannot be explained by sex differences in free drug plasma concentrations. The lower incidence of anticancer drug-induced heart-corrected QT (QTc) interval prolongation in male compared to female patients, for instance, may in part be due to testosterone's accelerating effect on ventricular repolarisation [63]. Androgen-deprivation therapy used for prostate cancer treatment may increase risk of Torsades de Pointes by prolonging QTc, as observed in a decade-long prospective study [63]. In another study, longer QTc measurements and more frequent prescribing of QT-prolonging drugs in women increased their odds of being women excluded from clinical trials of cancer therapies [64].

### 2.3 Sex differences in pharmacokinetics and adverse reactions to anticancer drugs

One of the key aims of early-phase trials is to characterise the pharmacokinetic (PK) profile of investigational drugs, as this informs dose and dose scheduling prior to registrational studies. Sex-based differences in body composition and organ function contribute significantly to pharmacokinetic disparities (Table 1). Women typically have approximately 10 % higher body fat, greater plasma volume and greater organ perfusion compared to men, impacting both drug distribution and onset of action [65,66]. Several pharmacokinetic analyses demonstrate that men generally have higher elimination capacities across multiple classes of anticancer drugs (Table 2) [[67], [68], [69], [70], [71], [72], [73], [74], [75], [76], [77], [78], [79], [80]]. This is likely influenced by sex-specific variations in the expression of drug-metabolising enzymes resulting from genetic polymorphisms in cytochrome P450 (CYP) isoforms [81] and the fact that renal function is approximately 20 % higher in males, increasing the rate of renal clearance [65]. Drug metabolism can often be estimated by basal metabolic rates, and men generally maintain a higher basal metabolic rate compared to women across all age groups. Conversely, CYP3A4, responsible for metabolising around half of all commercially available drugs, exhibits 25 % higher activity in women [82]; demonstrating the complex relationship between sex and drug elimination. The faster CYP3A4-mediated *N*-dechloroethylation to toxic metabolites in women receiving ifosfamide may contribute to the higher rates of neurotoxicity seen [83].

**Table 1:** Physiological differences in women compared to men and their impact on drug pharmacokinetics.

Parameter	Physiological differences in women compared to men	Pharmacokinetic impact in women
Percentage body fat	Higher	Slower processing of drugs due to less metabolically active FFM; higher V and accumulation of lipophilic drugs
Total body water	Lower	Variations in body composition affect drug distribution; lower V and higher plasma concentrations of hydrophilic drugs
Heart rate	Higher resting heart rate and longer QTc intervals	Higher risk of cardiac arrhythmias
Gastric motility	Lower gastric motility	Delayed drug absorption
Stomach pH	Higher gastric pH	Reduced absorption of acidic drugs
Hepatic enzymes	Different expressions of CYP450; higher expression of CYP3A4 and lower CYP2D6 and CYP2E1	Variations in drug metabolism rates
Renal blood flow	Lower renal blood flow	Slower excretion of drugs

Abbreviations: CYP, cytochrome P450; FFM, fat-free body mass; V, volume of distribution.

**Table 2:** Population pharmacokinetic analyses of selected anticancer drugs with sex differences in clearance.

Class, drug name	Indication	Study participants (n)	% Female	Variability in CL (CV%)	Relative changes in women vs. men	
**Alkaloids**						
Paclitaxel [67,68]	Solid tumours	319	50		CL	−30 %
Irinotecan [[69], [70], [71]]	Colorectal cancer, pancreatic adenocarcinoma	125	46	47 %	CL	−30 % to 38 %
**Alkylating agents**						
Temozolomide [72,73]	Glioblastoma multiforme, Malignant glioma	480	37	5–10 %	CL	−19 to 27 %
Melphalan [74]	Multiple myeloma, melanoma	64	66	52 %	CL	−19 %
**Angiogenesis inhibitors**						
Aflibercept [75]	Colorectal cancer	1506	49	31 %	CLfu	−16 %
Bevacizumab [76,77]	Solid tumours	2050	46	26 %		−14 % to −27 %
**Antimetabolites**						
5-Fluorouracil [78,79]	Solid tumours	116	36	22–40 %	CL	−14 % to −27 %
**Monoclonal antibodies**						
Rituximab [80]	Non-Hodgkin's lymphoma, chronic lymphocytic leukaemia	29	45	19 %	CL	−21 %

Abbreviations: CL, total clearance; CLfu, clearance of the unbound fraction; CV%, coefficient of variation.

Drug classes are presented in **bold.**

Sex differences also extend to the absorption and distribution of drugs. Lipophilic drugs, for example, generally have a larger volume of distribution in women, whereas the opposite holds true for water-soluble drugs [43]. Sex-based physiological variations in the gastrointestinal tract can also affect the tissue exposure of orally administered drugs [84]. Contrary to their presumed inert nature, some chemical excipients used in pharmaceutical dosage forms have recently been associated with a range of adverse reactions, from mild hypersensitivities to life-threatening reactions, sometimes in a sex-dependent manner [[85], [86], [87]]. Understanding these differences will become increasingly important as the number of pharmaceutical biosimilars of orally active agents increases, as dose interruptions or discontinuation due to adverse effects can have serious implications for patients with cancer.

Despite the fundamental understanding in clinical pharmacology that drug effects are produced by circulating concentrations rather than the administered dose, most drugs are evaluated in phase 1 trials and subsequently approved based on a patient's weight or body surface area (BSA). The consequences of this “one-size-fits-all' approach are far from abstract: a study of over 23,000 patients (38 % women) in phase 2 and phase 3 oncology clinical trials indicated that female sex was associated with a 34 % higher risk of experiencing severe toxicity from systemic cancer therapies [88]. Though insufficiently studied, available data clearly show that women are more susceptible to severe adverse drug reactions, including to objectively measured haematological toxicities (Table 3) [66]. 89 90–105 This issue is exacerbated by the notable underrepresentation of women in phase 1 dose-finding trials [106,107], making established dosages less generalisable across both sexes. Indeed, of the 10 drugs recalled from the US market between 1997 and 2000, eight posed greater health risks to women [108]. Moreover, a review of 86 drugs approved by the US Food and Drug Administration (FDA) revealed that 88 % showed elevated blood concentrations and longer elimination times (measured by C_max_ and AUC) in women compared to men when administered at standard doses [4].

**Table 3:** Sex differences in side effects and efficacy of anticancer drugs for non-reproductive cancers registered by the FDA in 2021 and 2022 [89].

Drug	Indication	Clinical trial phase (Clinical Trials.gov ID)	Study participants (n)	% Female	Grade 3–4 AEs (%)		ORR % (95 % CI)		Progression or death events (%)		HR (95 % CI) of treatment vs. control arm	
					M	F	M	F	M	F	M	F
Tremelimumab-actl [90]	Unresectable hepatocellular carcinoma in combination with durvalumab	Phase 3 (NCT03298451)	782	15	53	60	–	–	65^a^	77^a^	0.73 (0.61, 0.88)	1.02 (0.67, 1.56)
Tebentafusp-tebn [91]	Unresectable or metastatic uveal melanoma	Phase 2 (NCT03070392)	378	50	86	90	–	–	38^a^	31^a^	0.48 (0.31, 0.75)	0.57 (0.35, 0.94)
Mosunetuzumab-axgb [92]	Relapsed or refractory follicular lymphoma	Phase 1/2 (NCT02500407)	90	39	49	37	76	86	–	–	–	–
Futibatinib [93]	Intrahepatic Cholangio-carcinoma with FGFR2 gene fusions or other rearrangements	Phase 1/2 (NCT02052778)	103	56	73	79	38 (24, 54)	45 (32, 59)	–	–	–	–
Opdualag (Nivolumab and relatlimab-rmbw) [94]	Unresectable or metastatic melanoma	Phase 2/3 (NCT03470922)	714	42	79^b^	85^b^	–	–	47^c^	57^c^	0.68 (0.52, 0.89)	0.88 (0.65, 1.19
Teclistamab-cqyv [95]	Relapsed or refractory multiple myeloma among adults who have received at least four specific lines of therapy	Phase 1 (NCT03145181) Phase 2 (NCT04557098)	110	44	97	86	63	60	–	–	–	–
Mobocertinib [96]	Locally advanced or metastatic EGFR exon 20 insertion-positive NSCLC	Phase 1/2 (NCT02716116)	256	66	58	68	29 (13, 42)	29 (19, 41)	–	–	–	–
Tivozanib [97]	RCC	Phase 3 (NCT02627963)	350	27	65	71	–	–	69^d^	73^d^	0.64 (0.48, 0.86)	0.72 (0.44, 1.19)
Sotorasib [98]	NSCLC	Phase 1/2 (NCT03600883)	204	55	55	55	42 (30, 55)	31 (20, 44)	–	–	–	–
Melphalan flufenamide [99]	Refractory multiple myeloma	Phase 2 (NCT02963493)	157	43	94	97	23 (14, 34)	31 (18, 45)	–	–	–	–
Amivantamab-vmjw [100]	EGFR exon 20 insertion-positive NSCLC	Phase 1 (NCT02609776)	129	61	32	47	45 (28, 64)	35 (22, 51)	–	–	–	–
Asciminib [101]	Philadelphia chromosome-positive CML with T3151 gene mutations	Phase 1 (NCT02081378)	48	48	60	64	44^e^ (28, 62)	33^e^ (8, 70)	–	–	–	–
Tepotinib [102]	NSCLC	Phase 2 (NCT02864992)		52	21	22	44 (28, 62)	42 (25, 61)	–	–	–	–
Infigratinib [103]	Cholangio-scarcoma	Phase 2 (NCT02150967)	108	38	66	66	27 (14, 43)	21 (12, 33)	–	–	–	–
Umbralisib [104]	Follicular lymphoma and MZL	Phase 2/3 (NCT02793583)	221	43	17	27	52	47	–	–	–	–
Loncastuximab tersine-lpyl [105]	Large B-cell lymphoma	Phase 2 (NCT03589469)	145	41	50	13	50	47	–	–	–	–

Abbreviations: ALL, acute lymphoblastic leukaemia; AML, acute myeloid leukaemia; AE, adverse event; CI, confidence interval; CML, chronic myeloid leukaemia; FGFR2, fibroblast growth factor receptor 2; HR, hazard ratio comparing treatment of interest and comparator arm(s); LL, lymphoblastic lymphoma; MZL, marginal zone lymphoma; n, number of participants; NHL, non-Hodgkin lymphoma; NSCLC, non-small cell lung cancer, ORR, objective response rate; OS, overall survival; RCC, renal cell carcinoma.

a Percentage of deaths from any cause, calculated from the time of randomisation to the data cut-off date.

b All grades of adverse events.

c Percentage of progression (per RECIST v1.1 criteria) or death from any cause events, calculated from the time of randomisation to the data cut-off date.

d Percentage of progression events (per RECIST v1.1 criteria), calculated from the time of randomisation to the data cut-off date.

e Data presents subgroup analyses of major molecular response (BCR-ABL1IS ≤ 0.1 %) by 24 weeks.

In spite of these significant differences and the heightened focus on precision medicine, systemic cancer therapies still lack sex-specific dosing recommendations. The high attrition rates following phase 2 oncology clinical trials are of particular concern to clinical trial sponsors. The probability of success for phase 1 and phase 2 oncology clinical trials is only 58 % and 33 % respectively [109], with unfavourable pharmacokinetic profiles often cited as the main reason for discontinuing further development of drug candidates [110]. Clearly, accurately predicting and addressing biological differences, including sex, during the initial phases of clinical development is a critical concern for patients, healthcare providers, regulatory bodies and clinical trial sponsors.

## 3 Considerations towards the inclusion of sex in early-phase trials

**Fig. 1:**
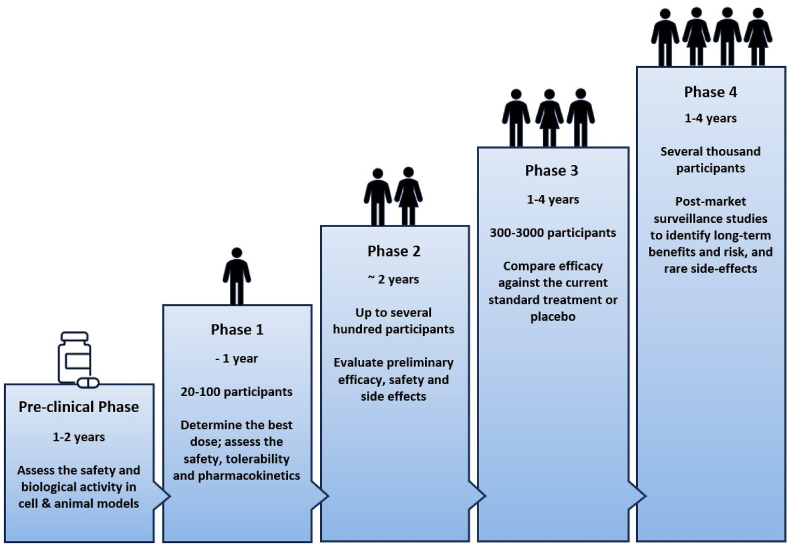
Phases of cancer drug development. This figure outlines the standard progression of anticancer drugs through the pre-clinical and clinical phases of development, detailing the typical durations, participant numbers and objectives of each phase.

Despite the increasing recognition of the influence of sex on the therapeutic response to anticancer drugs, early-phase clinical trials predominantly aim to characterise drug effects and establish dosing regimens for aggregate patient populations (Fig. 1). Investigators seeking to account for sex differences confront multiple challenges, such as questions concerning clinical trial design, dosage selection, and data collection, analysis, and interpretation. Given the profound impact of sex on therapeutic outcomes, adopting a multifaceted strategy is essential to effectively incorporate these differences into early-phase trials. In this context, we outline key areas for consideration for drug development in oncology.

### 3.1 Optimising the dose-finding paradigm

Traditionally, the approved dosing of anticancer drugs was often based on empirical, small-scale studies rather than rigorously designed trials with valid a priori assumptions. In the current paradigm of phase 1 clinical trials, including those using Bayesian models, the primary objective is to identify the MTD, often determined by administering increasing doses until a predefined proportion of patients experiences a dose limiting toxicity, thereby assuming that higher doses yield greater efficacy [111]. However, this approach has several limitations, namely, it fails to establish appropriate dose adjustments based on individual differences, such as sex, and often limits dose determination to a small cohort of heavily pre-treated patients [112]. The dose-response relationships of anticancer drugs are rarely well defined, particularly for targeted agents and immunotherapies as opposed to cytotoxic chemotherapies. In addition, the assumption that the dose selected will be optimal for other populations may well be incorrect. Indeed, analysis of phase 1 and 2 clinical trials has found patient characteristics have greater predictive value than dose for the toxicity of several cancer therapies [113]. Additionally, most new anticancer drugs targeting specific molecular pathways achieve maximum efficacy at doses lower than the MTD [[114], [115], [116]]. For these agents, dosing at the MTD is often inappropriate, as higher doses may cause off-target effects, increased toxicity, dose interruptions, and diminished compliance without improved efficacy [117]. This practice has led to the marketing of several drugs at non-optimal doses – including ceritinib, dasatinib, niraparib, ponatinib, cabazitaxel, and gemtuzumab-ozogamicin – necessitating further post-market adjustments [118]. In 2021, the decision by the FDA to mandate further post-market studies to refine the dose of the sotorasib – a first-in-class KRAS G12C inhibitor for NSCLC – as a prerequisite for its accelerated approval, signifies a shift for many in the regulatory scrutiny of oncology drug dosing [119].

Outside oncology, drugs are typically evaluated in randomised dose-ranging trials to assess the broader impact of varying doses on both efficacy and toxicity. In an exemplar case, the multi-kinase inhibitor nintedanib has been developed for both idiopathic pulmonary fibrosis and NSCLC: the former dose being selected based on a randomised dose-ranging phase 2 trial [120], whereas the higher oncology dose was selected based the MTD alone [121]. Recognising these issues, the FDA's Oncology Centre of Excellence launched Project Optimus in 2021 to reform the dose-selection paradigm for oncology drugs [112]. Subsequent guidance recommends conducting multiple dosage comparisons in randomised dose-ranging trials, potentially extending into registrational trials [117]. In this scenario, phase 1 trials would aim to establish a recommended dose range based on the totality of available data – including safety, tolerability, efficacy, pharmacokinetic and pharmacodynamic data. Dose determination could then extend into well-powered randomised dose ranging trials [117,122]. Trials should be flexible enough to allow separate cohorts of patients to enrol as needed, as it may be needed to define separate recommended doses based on patient or disease characteristics. This framework recognises the heterogeneity in patient responses, allowing for post-market dose individualisation that is supported by more extensive clinical trial data without challenging the drug's established therapeutic window. Although these new guidelines do not explicitly address sex as a biological variable, they highlight the need to consider the impact of patient populations on optimal drug dosing.

Dose modifications based on sex are not novel concept. For instance, a Swedish study demonstrated significantly improved survival outcomes in elderly women treated with rituximab for lymphoma [123], attributed to their reduced drug clearance and increased exposure [124,125]. Following this, the SEXIE-R-CHOP-14 trial investigated whether increasing the dose of rituximab in men aged 61–80 years with diffuse large B-cell lymphoma to 500 mg/m^2^ while treating women with the standard dose of 375 mg/m^2^ could improve outcomes [126]. Although prematurely terminated due to lack of funding, this academic trial showed that increasing the dose of rituximab in elderly men improved progression-free survival by 32.5 % (P = 0.039), with a trend towards better overall survival, demonstrating the feasibility and potential for sex-specific dose adaptations.

### 3.2 Fat-free body mass as a novel dosing parameter

Men and women have different body compositions, and this can influence drug metabolism. Men generally a higher percentage of metabolically active fat-free body mass (FFM)—accounting for approximately 80 % of body mass compared to 65 % in women of the same height and weight [127]. Men also tend to have more visceral fat and women have more subcutaneous fat [128]. Cytotoxic chemotherapy is typically dosed based on BSA using derived formulas to normalise drug exposure due to their characteristic narrow therapeutic range. However, the estimation of their model coefficients has been questioned: a comparison of 25 BSA formulas has shown the calculated BSA value can vary by 0.5 m^2^, depending on the specific formula used [129]. Similarly, dosing according three BSA bands (<1.7 m^2^, 1.7 m^2^ – <1.9 m^2^ and ≥1.9 m^2^) yielded comparable target AUC values as dosing according to the individual-BSA-based calculation for the cytotoxic agents cisplatin, docetaxel, doxorubicin, irinotecan, paclitaxel and topotecan [130]. This inexactitude likely results from the fact that BSA-based dosing assumes that drug distribution is directly proportional to BSA and does not account for important differences in body composition or pharmacokinetics. The Du Bois formula, developed in 1916 for calculating basal metabolic rate and only applied several decades later to oncology, remains the most used formula for BSA-based dosing. That only nine male subjects were used in its derivation does not appear to be well known, nor does it justify its ubiquitous application [131].

Compared to BSA and body mass index (BMI), FFM serves as a better estimate for metabolically active body mass. FFM, which incorporates a sex coefficient and thus better reflects sex differences in renal and metabolic clearance, has been shown to more accurately predict drug clearance rates [127,132]. While magnetic resonance imaging (MRI) is the gold standard for measuring body composition, a single abdominal computed tomography (CT) scan without contrast enhancement of the L4-L5 region provides an economically feasible and excellent alternative [133,134]. The utility of FFM as a dosing parameter were demonstrated in several studies. A meta-analysis of 242 metastatic renal cell carcinoma patients, which found low skeletal muscle index was associated with a significantly higher rate of dose-limiting toxicity in patients receiving the tyrosine kinase inhibitors sunitinib and sorafenib [135]. In a separate a retrospective analysis of 107 children with lymphoma and rhabdomyosarcoma found high skeletal muscle density, estimated by routine CT scans at diagnosis, was associated with lower risk of grade 4 haematological toxicity with cytotoxic chemotherapy due to changes in biodistribution [136]. Furthermore, a prospective study of 60 colon cancer patients receiving adjuvant 5-fluorourical found a threshold of 20 mg/kg lean body mass for developing toxicity [137]. Given these findings, FFM offers a promising alternative dosing parameter for early-phase studies to more precisely define the therapeutic range across both sexes.

### 3.3 Integrating sex-specific variables in PBPK modelling and simulation

Physiologically based pharmacokinetic (PBPK) modelling serves as a valuable tool to address issues of drug attrition attributed to poor pharmacokinetic behaviours in early drug development [138]. These models allow for the targeted integration of sex-specific physiological and pharmacokinetic parameters to better predict inter-individual variability in drug response. For example, absorption values, rate constants, scaling factors, and enzyme or transporter activity coefficients can be calibrated to mirror sex-specific physiological differences [139]. Parameter sensitivity analyses can be tested to understand if sex-specific parameters are required to accurately predict drug response between men and women, with the goal of constructing a model that provides insight into the optimal dose for both sexes [140,141]. Indeed, regulatory agencies such as the FDA and European Medical Agency have encouraged pharmaceutical companies to use PBPK modelling to understand drug response since the 1990s [142,143].

The development of sex-specific models requires quantitative data for the physiological sex differences; however, current literature often presents sex differences in a relative manner (e.g., “males > females”) [144]. Here, machine learning (ML) and artificial intelligence (AI) can offer significant contributions, particularly in the handling of large and complex data sets [145]. Drug development is increasingly being guided by Big Data, and AI has already been used in various aspects of drug development, including to assess patient response to cancer therapies [146], drug-drug interactions [147] and adverse drug reactions [4,148]. However, its application in predicting sex-specific pharmacokinetics and pharmacodynamics is still grossly underutilised, and many ML models fail to account for sex as a variable which could risk sex-biased outcomes [149]. The predominance of white male subjects in genome-wide association studies exacerbates this issue, particularly as molecular biomarkers are increasingly being used for clinical trial enrichment [139]. Furthermore, the FDA has recently approved the use of several digital biomarker devices to measure a range of symptomatic and prognostic markers in clinical trials [150]. Ramsey et al. demonstrated that up to 56 % of serum biomarkers show sex-based variation, concluding that sex and female hormonal status should be reported when collecting biomarker-related data [151]. To realise the potential of these insights into optimised therapeutic strategies, population-based models must be both formulated and validated with sex variables in mind.

### 3.4 Methodological challenges of including sex as a biological variable

Despite significant progress towards personalised treatment in oncology, reservations persist amongst the research community about the implications of analysing the sexes separately, particularly in early-phase clinical trials given their modest sample size [152]. Critics of sex analysis claim that designing and conducting scientifically rigorous clinical trials with enough statistical power to detect sex differences is both cost and time prohibitive [9]. However, researchers are beginning to recognise the importance of examining sex differences in the pharmacology of anticancer drugs to address disparities in therapeutic outcomes. The oncology field has already embraced the exploration of rare tumour types and the subdivision of tumours based on their histology or molecular profiles. In this context, incorporating sex as variable should not be regarded as a barrier; rather, it is an attainable and essential objective that aligns with the broader goal of personalised medicine. Indeed, sex is a good stratification candidate since each group corresponds to a large fraction of the total sample population [153]. While early-phase trials may not be designed for formal hypothesis testing, exploratory subgroup analyses can be cautiously interpreted to generate hypotheses for future research [154], and emphasising confidence intervals over p-values can enhance the interpretive validity of results [155]. Methods to correct for multiplicity, such as Bonferroni correction, can mitigate the risk of type 1 errors [13]. For this reason, the a priori specification of tests for selected adverse events by sex is considered important for improving the interpretation of safety results.

Bayesian statistical methods offer a strategy for addressing concerns about subgroup analysis. These methods allow for the integration of prior knowledge with current trial data, thereby providing a more robust foundation for assessing sex-related outcomes [13] and reducing the sample size requirements of traditional frequentist analyses [156,157]. In addition, Bayesian approaches allow for adaptive trial designs that can be adjusted based on interim findings, increasing the likelihood of identifying meaningful effects [156]. However, some researchers caution that reliance on prior assumptions can substantially influence the study's conclusions [158]. This issue gains particular resonance in light of the recommendations form the Institute of Medicine Committee on Women's Health Research, which advocates for the assumption of sex-based differences in medical research unless there is compelling evidence to suggests otherwise [159].

### 3.5 Addressing gaps in sex-based pharmaceutical research and regulation

The regulatory landscape relating to the equal representation and reporting by sex in biomedical research has changed significantly over recent decades. Efforts to counteract male bias in biomedical research gained traction with the 1993 NIH Revitalization Act, which moved from a stance of excluding to recommending the inclusion of women in phase 3 clinical trials [160]. Multiple funding organisations, such as the US National Institute of Health (NIH) and more recently the Medical Research Council [161] and Cancer Research UK [162], now require the integration of sex in experimental designs. Nevertheless, pre-clinical research often defaults to male-only studies, owing to unfounded concerns about hormonal cycles in females and other impediments such as the perceived complexity and cost implications if sex analysis is included [8,9].

Major regulatory bodies in the US, Europe, and Canada, whose approvals often influence other countries, have made some initial efforts to mandate the inclusion of sex analysis in pharmaceutical regulations though there remains significant scope for improvement [14]. For example, the International Council for Harmonisation continues to consider women as a special subgroup, lacking standalone guidelines for their inclusion [163]. This fragmentation extends to academic publications and industry practices, where sex-disaggregated data concerning drug efficacy, safety, and toxicity are inconsistently reported [164,165], and sex-specific information seldom appear on product labels [166]. Despite improvements in the practice of including both sexes since the 2016 NIH requirements, sex-disaggregated reporting has remained stagnant across multiple high-impact journals and disciplines. In eight of nine disciplines across 720 articles in 34 high-impact journals, no changes have been seen in sex-disaggregated reporting and analysis [9]. The Sex and Gender Equity in Research (SAGER) guidelines provide a roadmap for journal editors to reject manuscripts that do not report or discuss sex considerations [167]. However, without systemic enforcement of these guidelines, evidence gaps in the understanding of sex-specific health needs will persist.

Women continue to be severely underrepresented in clinical research, particularly phase 1 clinical trials [168,169]. Current guidelines suggest that, at a minimum, women should be represented in clinical trials in proportion to their prevalence in specific health conditions [163]. Yet this target falls short in some cancers, and a focus solely on prevalence will not capture sex differences in the progression of disease or mortality. In 2019, for instance, the FDA approve erdafitinib for FGFR + advanced urothelial carcinoma based on a cohort of 87 participants which was comprised of only 18 (21 %) women [170]. The justification provided was that men are three to four times more likely to develop urothelial carcinoma, despite women having poorer survival outcomes even with alternative interventions such as radical cystectomy [171,172].

To redress these shortcomings, regulatory agencies should explore a range of strategic incentives and penalties. Drawing lessons learned from drugs developed for rare diseases [173] and paediatric populations [174], these may include research design support, fee waivers, expedited review, brief patent extensions and tax credits. The introduction of regulatory penalties for insufficient sex-disaggregated evidence could complement these incentives as part of an integrated international strategy. Additionally, agencies should mandate the reporting of sex differences on drug labels and make all sex-disaggregated data submitted by pharmaceutical companies publicly available. Nevertheless, effective regulation can only be achieved through engaged collaboration with industry stakeholders.

## 4 Conclusion

Incorporating sex as a biological variable in pharmaceutical research and regulation is essential for optimising treatment outcomes for both men and women. Such inclusion will strengthen scientific rigour, encourage innovation by identifying novel mechanisms, and promote health equity amongst patients with cancer. Given the consensus about the existence of sex differences in cancer incidence, treatment response, and survival, overlooking sex as a biological variable from the earliest stages of drug development would be both scientifically untenable and ethically irresponsible. As the oncology community intensifies its focus on personalised medicine, critical revisions are needed in early-phase clinical trial paradigms to ensure optimal dosing strategies for both sexes. This will not only improve outcomes but also reduce toxicity to anticancer drugs, signifying a promising avenue for enhancing the efficacy and safety of cancer treatments.

## CRediT authorship contribution statement

**Lydia Sutherland:** Writing – original draft, Writing – review & editing. **Louise Carter:** Conceptualization, Supervision, Writing – review & editing.

## Declaration of competing interest

The authors declare that they have no known competing financial interests or personal relationships that could have appeared to influence the work reported in this paper.
